# Decomposition of partially submerged remains: a study on the reliability of insect colonisation for PMI/PMSI estimation

**DOI:** 10.1007/s12024-024-00871-y

**Published:** 2024-08-14

**Authors:** SK. Bray, XA. Conlan, ML. Harvey

**Affiliations:** School of Life and Environmental Sciences, Faculty of Science, Engineering and Built Environment, Deakin University, Geelong, Australia

**Keywords:** Aquatic decomposition, Entomology: insect succession, Partial submersion

## Abstract

The terrestrial decomposition of remains and associated insect colonisation have been highly researched, and recently studies have expanded to investigate the aquatic decomposition of remains. However, there are instances where remains may experience both terrestrial and aquatic conditions simultaneously due to partial submersion in tidal areas, or influx or efflux of water caused by flood or drought. Decomposition and post-mortem interval (PMI) research to date has focused on remains wholly exposed to either terrestrial or aquatic environments, with limited consideration of dual simultaneous exposure. This study was conducted in artificial lentic environments to ascertain how simultaneous zones of terrestrial and aquatic environments on a single body may impact decomposition. Three trials were completed over a period of 12 months, with each trial consisting of 12 stillborn piglets; three partially submerged head exposed, three partially submerged abdomen exposed, three fully submerged aquatic controls and three terrestrial controls. Decomposition stage and rate were inferred from physical characteristics and insect activity. The decomposition rate of the exposed region of each piglet was significantly faster than the submerged region. The exposed zone of each was colonised by insects and reached skeletonization, whereas the submerged zone without orifice exposure had no insect activity and had a significantly slower decomposition rate. This indicated the ability to utilise terrestrial entomological approaches to estimate a minimum PMI for the exposed portion of the remains. However, without the ability to determine the amount of time the remains may have been submerged for, this estimation represents only a minimum PMSI, with the possibility the remains were submerged for a period of time without insect access and colonisation.

## Key points

• Decomposition rate differed significantly between the aquatic region and terrestrial region.

• Insect activity on the exposed/terrestrial region of the piglets was a key contributor to the exposed region reaching skeletonization, in contrast to the aquatic region of the partially submerged piglets which showed little to no insect activity.

• Orifice access contributed to differences seen between partially submerged piglets, where piglets with heads exposed colonised earlier than piglets with abdomen exposed and head submerged.

## Introduction

Previous research has been focused on terrestrial decomposition and entomology as a post-mortem interval (PMI) estimation method for remains in forensic casework [1]. More recent case studies [2–5] have determined a high frequency of remains discovered in aquatic environments, but with little controlled research on aquatic decomposition there is a knowledge gap around aquatic decomposition and estimating post-mortem submersion intervals (PMSI) in aquatic cases. Most recent studies on aquatic decomposition have largely focused on large water bodies such as rivers [2, 3] and the ocean [4, 5], investigating factors in full submersion scenarios such as algal colonisation, aquatic decomposition patterns and scavengers. However, there is limited documentation of decomposition in environments such as tidal areas, shallow waters where remains are subject to both terrestrial and aquatic factors.

PMI is the term frequently used in terrestrial research and casework to refer to the time since death. In contrast, PMSI refers to the submersion interval of remains and is the term used in aquatic casework and research. A PMSI is established to determine the time the remains were submerged for but doesn’t consider the time the remains may have been outside of the water before submersion. This is an important distinction to consider when discussing casework. Further, in terrestrial cases there is the need to consider the pre-appearance interval (PAI) of insect colonisation as an unknown period, thus a minimum PMI is established (PMImin); this is considered the minimum amount of time the remains have to have been there for based on the time required for the insects to have reached that life stage/level of growth [8]. In an aquatic setting there may be the PAI to consider after remains have floated or become partially submerged, but there is also an additional unknown submersion period to account for.

Terrestrial decomposition follows a well-established pattern of stages: fresh, bloat, active decay, advanced decay and dry/skeletal [1]. These stages are accompanied by predictable characteristics and documented insect succession where Calliphoridae are primary colonisers in the fresh to active decay stages, followed by secondary dipteran colonisers and Coleoptera in active decay stages and towards the advanced decay and dry stages [6, 7]. In contrast, there are no true obligate necrophagous insects in aquatic environments [7, 8], with most insect activity relevant to aquatic decomposition being flying insects with access to the remains only during periods of bloat and float [9, 10]. Aquatic decomposition has its own defined decomposition stages, being submerged fresh, early float, floating decay, floating deterioration, floating remains, and sunken remains [10]. However, these stages are less predictable in onset and duration than terrestrial stages and are impacted by variables such as float and bloat pattern and orifice access for insects [11].

Recent aquatic studies have focused on complete submersion and use of the total aquatic decomposition score (TADS) [12, 13] as a method to determine PMSI. These studies were able to establish a PMSI using a combined approach of accumulated degree days (ADD) and physical morphology of remains, however presented large confidence intervals and in Dalal’s model a consistent overprediction of PMSI [13]. Further, this method has only been researched in complete submersion cases and cannot be directly applied with confidence to hybrid/partial submersion cases without further research.

Other studies have evaluated the use of terrestrial based PMI estimation methods such as insect activity in aquatic environments, stemming from Payne and King’s initial work on aquatic decomposition in 1972 [10]. Heo et al. [9] researched insect succession in aquatic decomposition using a man-made freshwater pond system. Though insect colonisation in this study was high, there was an unknown submersion interval of time and significant insect death due to the carcass sinking that determined this to be an inaccurate method to estimate PMSI. Bray et al. [11] also presented the lack of ability to use insects in aquatic cases as a result of an unpredictable submersion interval, emphasising a lack of predictable insect succession. Similarly, the difficulty in determining the unknown time period between initial submersion and floating remains adds further potential discrepancies for PMSI estimation. This unknown submersion period confounded with the pre-appearance interval (PAI) of insects to the now accessible corpse creates considerable potential error for PMI/PMSI estimations utilising insects.

This has highlighted the need for further studies into increasing the confidence of PMSI estimations, achievable through further research of aquatic variables, the adaptation of approaches, and combining approaches for increased accuracy. Several recent studies [4, 5, 9, 11, 14–16] have investigated aquatic decomposition of completely submerged remains, yet no studies have considered how partial submersion might result in dual zone scenarios with both aquatic and terrestrial components, outside of the float and bloat stages of aquatic decomposition. Methods such as TADS [13] are only applicable when the remains are entirely subject to an aquatic environment, otherwise discrepancies in physical characteristics will be unable to provide a reliable PMSI. Further, although entomology is well established in terrestrial casework for PMI estimations, without understanding the effect the proximity of water has on insect attraction and colonisation of partially submerged remains insects cannot be utilised in aquatic case work for PMI/PMSI estimations. When considering tidal, draught and flood environments where remains are likely to be only partially submerged, there needs to be an understanding established of the potential for remains to be segmented into distinct aquatic and terrestrial regions, if there is cross over between the two environments, and what this means for insect succession and the use of entomology for PMI estimation.

This study considered the potential of hybrid situations between terrestrial and aquatic decomposition, with the aim of establishing how the presence of two distinct decomposition environments affects the rate of decomposition of a single carcass comparatively. Further, this study aimed to establish a recommended approach for estimating PMSI in such scenarios.

## Method

This study was completed in Regional Victoria, Geelong, Australia (− 381,976, 144.2981) between late summer 2020 and spring 2021, and consisted of three trials with twelve stillborn piglets in each. The method follows closely to Bray et al. [11]; 9 × clean 100 L paddle pools (W 120 cm, L 120 cm, D 50 cm) were filled with tap water and placed in a large, fenced field in full sun 5 m apart. New pools and tap water were used for each trial, with water levels not refilled for the duration of each trial to decrease investigator interference. Though carcass size is a key factor in decomposition and carrion entomofauna [8], piglets were deemed an acceptable human model for this study, as it was preliminary study with a focus on comparison between the two environments of a carcass.

**Fig. 1:**
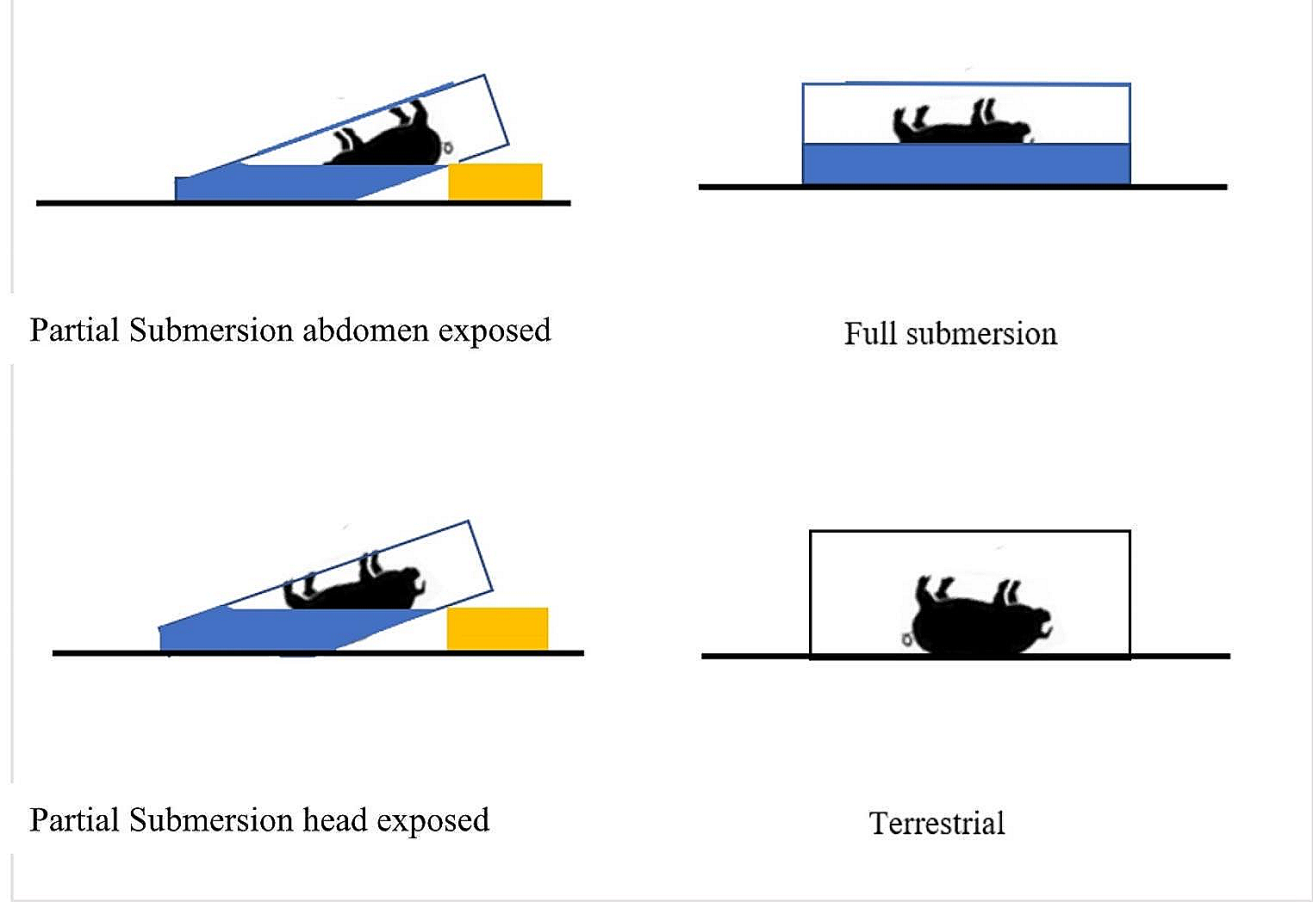
Field trial experimental design. three piglets in each condition: partial submersion head exposed, partial submersion abdomen exposed, terrestrial and full submersion. All piglets covered by mesh cage/wire (1 cm × 1 cm mesh)

Three piglets weighing between 1.5 and 2.5 kg each were placed in individual pools to act as aquatic controls, and three piglets were placed on the ground with a wire cage (1 cm × 1 cm wire mesh cage) over the top to prevent scavenging and act as terrestrial controls. The remaining six piglets were also placed in pools; however, the pools were place on a roughly 45 ° angle by stacking bricks under one side to produce an exposed/dry ‘terrestrial’ region and an aquatic region. Three of these six piglets were placed head out of the water and abdomen submerged, and the remaining three were placed head submerged and abdomen exposed as seen in Fig. 1. Each carcass had the unrestricted opportunity throughout the decomposition process of unimpeded movement. All containers had wire mesh (1 cm $\:\times\:$ 1 cm wire mesh) over the top to prevent scavengers but not preclude insect activity.

Changes to physical characteristics were recorded, with decomposition stages determined and refined by stages and characteristics described in Payne and King [10] and Van Daalan et al. [5]. Insect activity was noted and photographed every other day for a period of several weeks until the piglets had reached skeletonization (or partial skeletonization). As soon as new insect colonisation was observed, a sample of the larvae was taken to the lab for rearing and identification. Rearing was achieved using beef liver chunks as a food source in an incubator (Thermoline Scientific model no. TRHL-460-1-SD) set at 22 ⁰C and 40% humidity with a 12:12 photoperiod. Once emerged, the species was identified under a stereo microscope and recorded.

One Hygrochron temperature and humidity logger i-Button (DS1923-Maximintegrated) was secured with cable ties onto a terrestrial piglet cage in the field and one onto the mesh above one of the ponds. The i-Buttons were set to collect temperature data hourly then downloaded and viewed in OneWire Viewer, which was used to determine accumulated degree days (ADD) by averaging hourly temperature across each day (a 24-hour period) and accumulating the daily average [17]. An upper threshold was not considered in ADD calculations due to temperatures not reaching the upper limits required for calliphorid development. Similarly, a lower development threshold (LDT) was not considered when calculating ADD due to the ephemeral, unestablished micro system that has no pre-existing insects or aquatic life. Further, the water temperatures did not reach the lower thresholds of the common primary colonisers in the area and so an LDT was not considered. HOBO™ water temperature loggers were placed in two of the pools in each trial to determine any temperature differences between the two environments.

## Results

**Fig. 2:**
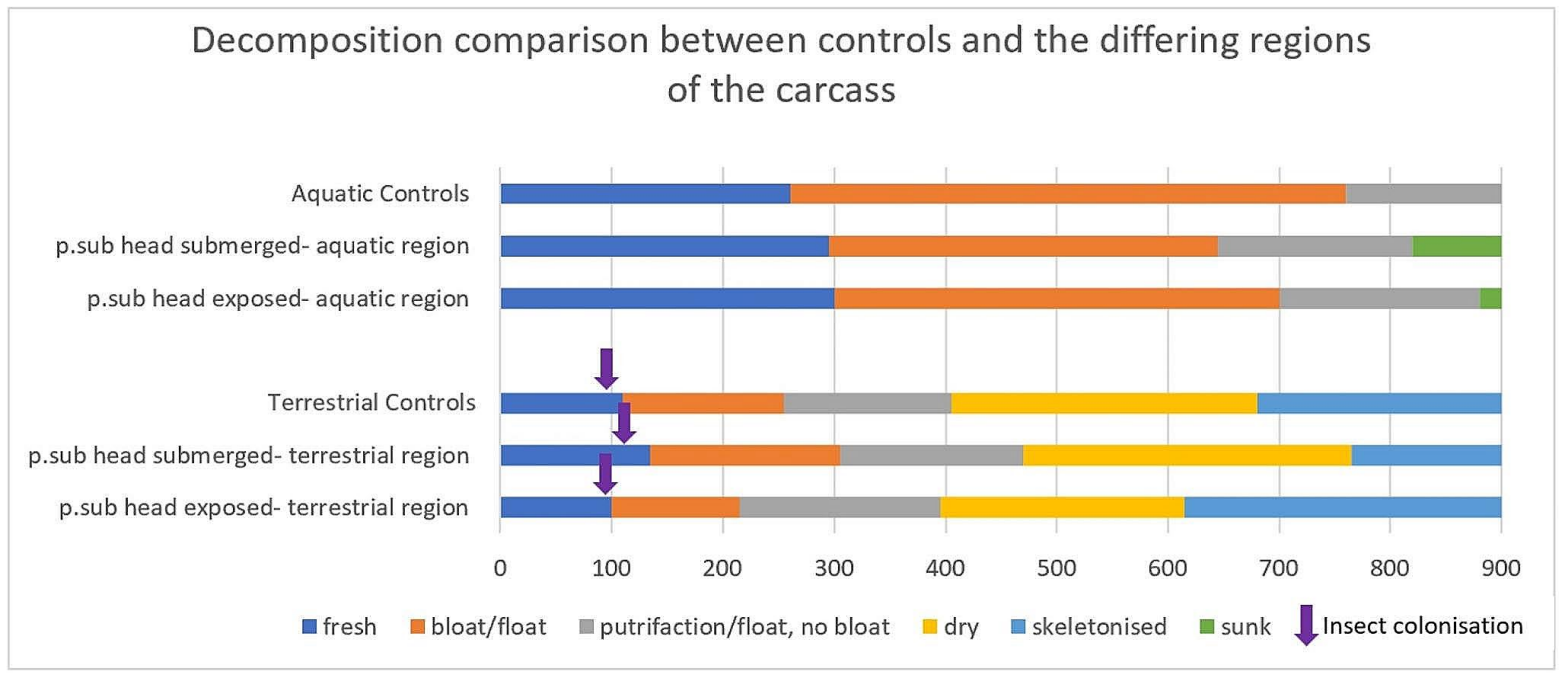
Decomposition rate and time of insect colonisation between conditions and environment zones. P.sub = partial submersion

Terrestrial control piglets across all trials followed the predicted pattern of decomposition established in previous research, progressing through all stages with associated insect colonisation and succession. Primary colonisers *Calliphora stygia* (Fabricius), *Calliphora augur* (Fabricius), and Diptera muscidae arrived on the remains within hours, followed by secondary dipteran colonisers *Lucilia sericata (*Meigen), *Chrysomya rufifacies* (Macquart) and Coleoptera from the active decay stages onwards. Conversely, fully submerged aquatic control piglets had little to no insect colonisation and varying lengths of time spent in the decomposition stages. While the terrestrial controls progressed through to dry or skeletonized within a few weeks, the fully submerged piglets were still in bloat or floating decay with no insect colonisation at the same timepoint. The decomposition rate and stage comparison can be clearly seen in Fig. 2 below.

**Fig. 3:**
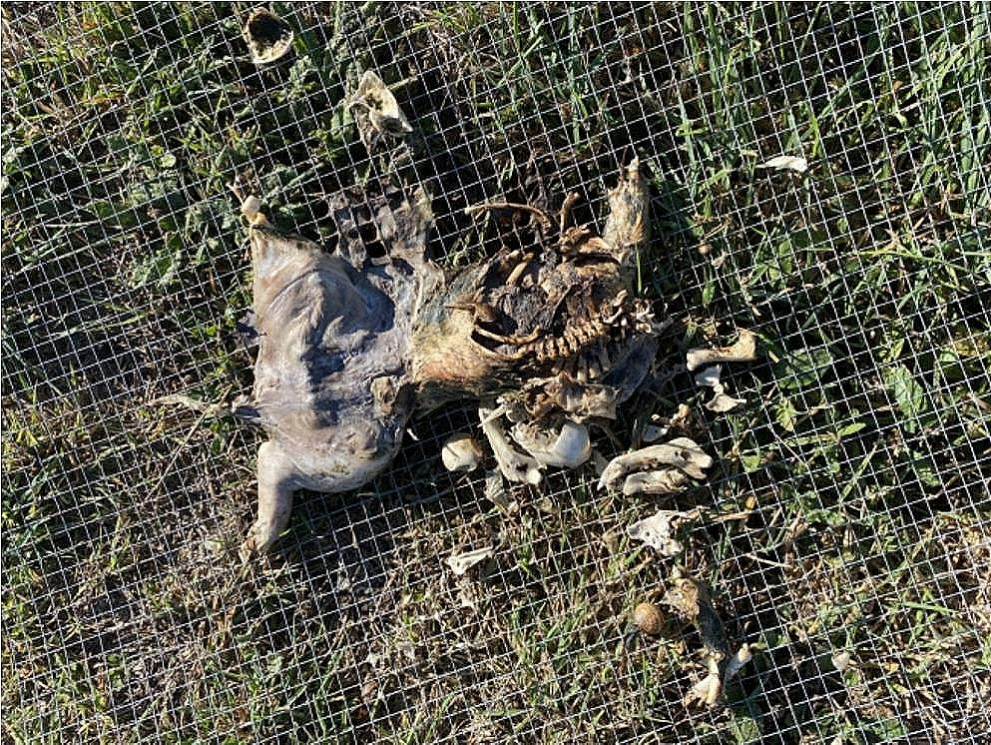
Abdomen submerged, head exposed piglet replicate removed from water after completion of trial, showing clear distinctions in decomposition between the two regions

**Fig. 4:**
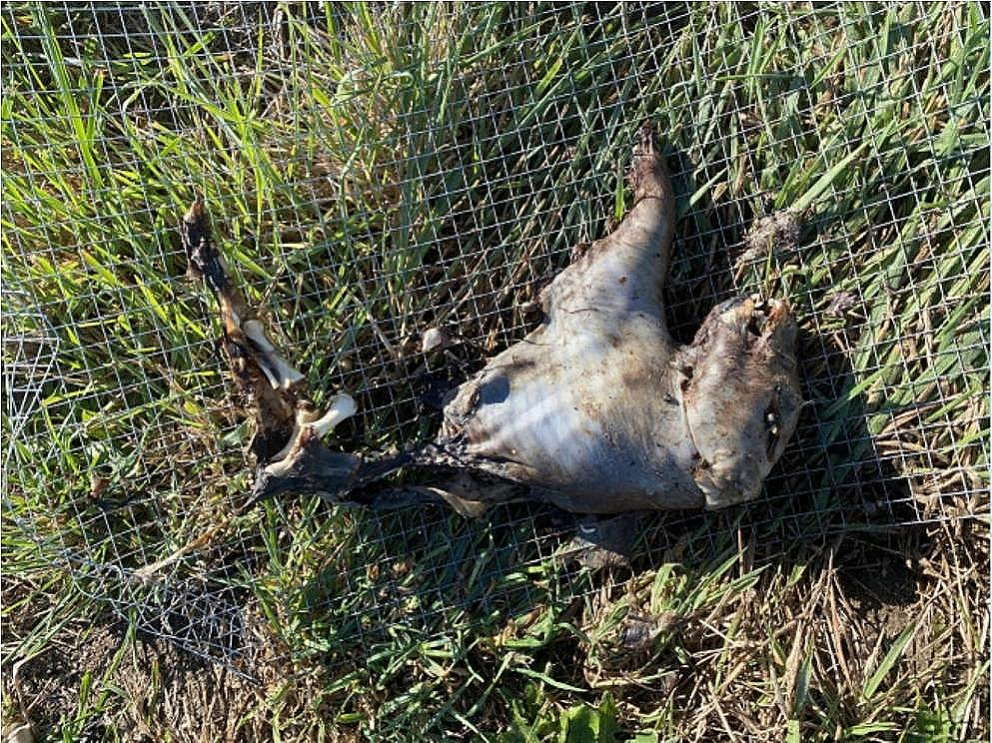
Head submerged, abdomen exposed piglet replicate removed from water after completion of trial, showing clear distinctions in decomposition between the two regions

Partially submerged piglets showed distinct delineation between terrestrial decomposition and aquatic decomposition at the water line. This was characterised by morphology, insect presence and decomposition stage, with the exposed/terrestrial region experiencing colonisation and active decay and the submerged region remaining fresh with no insect activity recorded. These distinct aquatic and terrestrial regions on the one carcass were seen with all replicates and across trials, with the terrestrial region progressing though active decay with insect colonisation to skeletonized while the aquatic region was still fresh or bloated. This was seen in both piglets with heads exposed and piglets with the abdomen exposed and head submerged, depicted in Figs. 3 and 4.

Further, though both head exposed/body submerged and head submerged/body exposed piglets had the same clear distinction between the terrestrial and aquatic environments, there were slight differences in the length of time it took piglets to be colonised by insects, as seen in Fig. 2. Piglets with their head exposed were colonised at the same time as the terrestrial piglets and several days before the piglets with abdomen exposed and head submerged, showing 3rd instar larvae after one week compared to the head submerged piglets showing only eggs and firsts around the anal orifice and between the hind legs. This trend continued, with head submerged piglets following the head exposed piglets two to three days behind in decomposition stages and progression.

Interestingly, during active decay with high larval activity on the terrestrial regions of partially submerged piglets, larvae were observed to be present and feeding only above the water line. Even when burrowed into the carcass and feeding from the inside the larvae showed a clear line of feeding that matched the water line, disregarding the submerged region of the piglets completely.

Coleoptera were also present in the pools, predominantly floating (dead) and consisting of Sylphidae, Histeridae, Elateridae and Trogidae, with their presence in the early stages of decomposition contrasting typical terrestrial succession. However, their presence in the water did not appear to impact colonisation on the terrestrial regions of the partially submerged piglets, which had insect colonisation follow succession patterns typical of terrestrial decomposition.

## Discussion

It was observed that discrete zones exist between terrestrial and aquatic environments on a single body experiencing partial submersion, as opposed to an overlapping or intermediate zone between the two environments. Partially submerged piglets in all trials followed previous literature for the submerged regions [10, 11], with little/no insect activity and significantly slower progression than terrestrial decomposition. Similarly, the exposed region of each piglet followed the terrestrial controls decomposition patterns colonising and progressing to skeletonized or dry within weeks. Three trials were conducted at different times of year; however, season was not observed to impact observations or time in decomposition stages between replicates significantly.

The delay in colonisation of the abdomen exposed piglets compared to the head exposed and terrestrial control piglets is likely due to variation in orifice exposure and access for insects. With the head exposed there are more orifices (eyes, ears, mouth) that are easily accessible and colonisable by insects. In contrast, the number of orifices decreases if the piglet is submerged headfirst, with only the anus accessible above the waterline in some cases. This observation has been detailed in previous literature concerning impacts of insect access/exclusion and orifice access studies [18, 19]. Thus, in the piglets with head submerged and abdomen exposed colonisation was delayed by two to three days when compared to the head exposed piglets. However, both regions when above the water were colonised by insects and progressed to dry/skeletonized alongside the terrestrial control piglets, with no discernible impact from the submerged region of the carcasses. The distinct line of separation between the two environments may have resulted from larval aversion to water [20–23]. Where there was enough food above the surface the larvae did not need to venture below the water level, resulting in a clear distinction between the two environments. Additionally, the discrepancy in time seen between colonisation of the partially submerged piglets could be due to the flies in the area preferentially selecting the head/face orifices over the single posterior orifice. It is possible the flies would have utilised the posterior exposed piglets more readily if there were no other easily available options in proximity.

The way in which the terrestrial/exposed zone of the carcass decomposed with no apparent impact from the aquatic region highlights the potential to separate a partially submerged carcass into two separate zones for a PMI/PMSI estimation. By considering the remains as two separate zones there is opportunity to employ either terrestrial PMI methods, aquatic PMSI methods, or a combination of to estimate PMI/PMSI. However, there needs to be further research investigating TADS, algal succession and other aquatic methods to ensure the reliable use of these in practical application. With no impact on insect succession from the proximity of water it is possible to utilise insects for terrestrial based PMI estimations with good accuracy. Yet, if remains have been subject to a tidal environment or influx/efflux of water that may have caused changes to the submersion/exposure of a carcass the reliability of insect colonisation will be impacted due to their inability to survive in aquatic environments [20–23]. Further studies investigating environments with influx/efflux of water would further the findings of this study, where contained water bodies without influx/efflux of water were utilised with a specific focus of determining how the presence of two distinct decomposition environments affects the rate of decomposition of a single carcass comparatively. Thus, the water in this study became more concentrated as decomposition progressed, with further studies able to consider influxes of water and tidal changes. In addition, while additional water was not added at any point throughout the trials after set up, the water levels through any season did not drop low enough to expose the submerged region of the piglets. Further, it is becoming increasingly clear there are surmounting errors associated with an unknown submersion period prior to floating remains. Hence, the estimation of PMI/PMSI for remains will always be a minimum estimation (PMImin), with this unknown time and PAI confounding into a potentially large error margin. Consideration into how this time period could be determined should be the focus for further research going forward.

A few studies have investigated insect/pupal survival in water, determining a significant decrease in survival correlating to the length of time submerged [7, 21–23]. This highlights further the unreliability of solely using insects for floating remains, or where remains have been subject to continued fluctuating water levels. Thus, an understanding of the water level fluctuations in individual forensic cases needs to be considered when determining the best method for PMI/PMSI; in cases with fluctuating water levels the use of aquatic methods may prove to be more useful (with further research), but with insects still providing a PMImin if present. Employing methods such as TADS or algal growth [7, 24] for the submerged zone in these cases may be able to provide a closer estimate of the minimum PMI (PMImin) compared to utilising insect activity, due the unknown pre-appearance interval (PAI) of the insects and unknown submerged time for fluctuating water levels. Alternatively, utilising both terrestrial and aquatic methods for partially submerged cases may be able to provide further accuracy, such as employing TADS or algal succession for the submerged region [7, 24], and entomology for the exposed region. In addition, further studies exploring partial decomposition in tidal conditions, and varying water types (such as chlorinated and brackish) would be beneficial.

Overall, the clear distinction between the two regions on a single carcass demonstrates the possibility of utilising insects in partial submersion cases for PMI estimations. Though proximity of the water had no effect on the decomposition rate of the exposed region of carcass, the use of insects only provides a PMImin with the need to consider the piglet orientation and varying water levels on the length of the unknown full submersion period. This is due to piglet orientation impacting orifice access to colonisers, and therefore will have an effect on the period of time before colonisation of the remains. Further, in cases where water level fluctuations have caused the remains to vary between partial and full submersion, an aquatic PMSI estimation approach such as TADS or algal succession may prove more reliable in addition to a PMImin from insects in forensic casework.

## Data Availability

All data is presented in the results section of this paper, no further data sets are provided. Raw data can be sourced from the primary author.
